# Supplementary material for: Mediterranean Pine Vole, Microtus duodecimcostatus: A Paradigm of an Opportunistic Breeder
Source: Animals (Basel). 2021 Jun 1;11(6):1639. doi: 10.3390/ani11061639 (PMC8228771; doi:10.3390/ani11061639)
Supplement: Supplementary file 1 [file animals-11-01639-s001.zip › Supp_figs_methods.pdf]

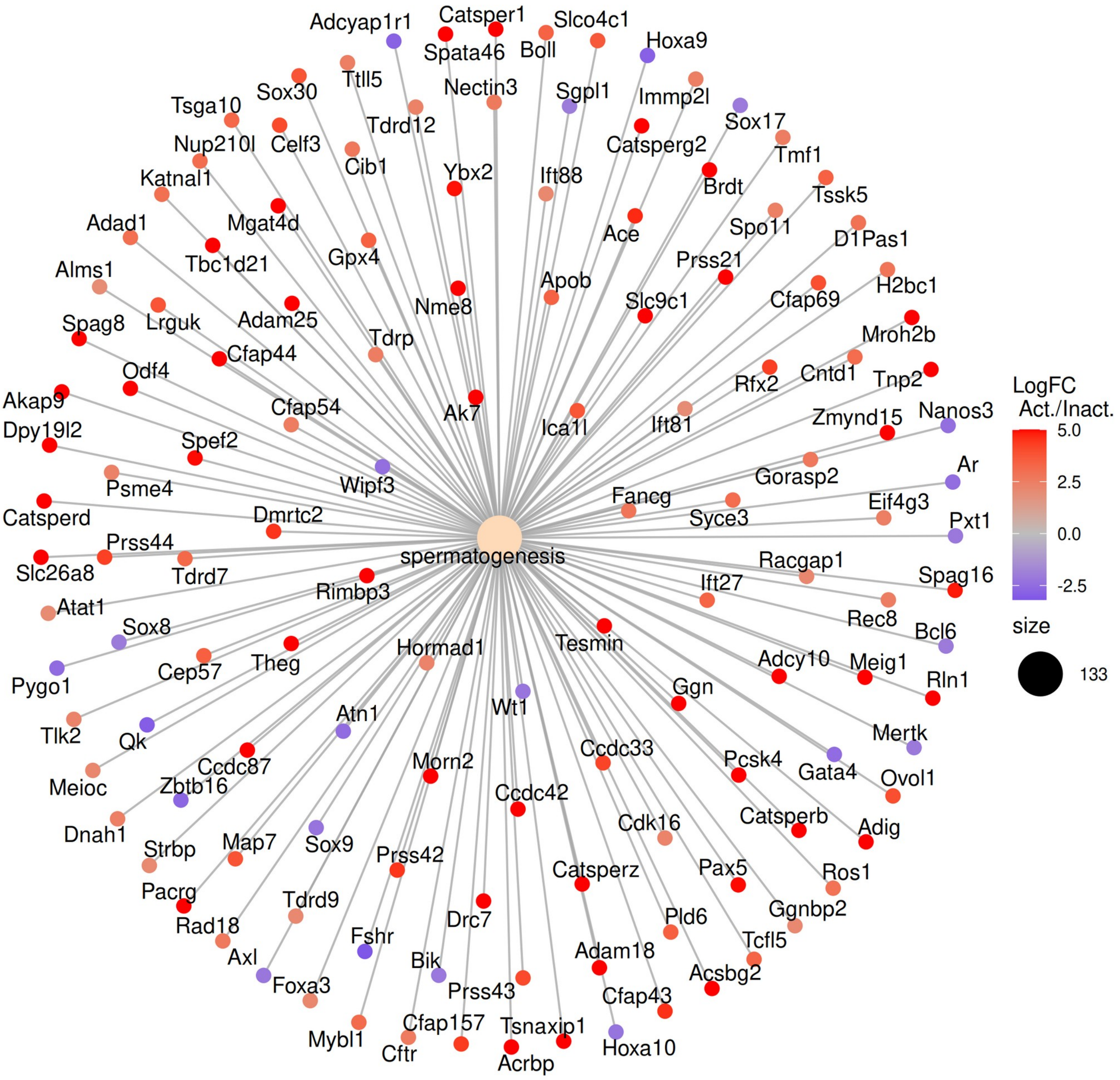

Supplementary Figure S1. Cnetplot of the GO term *spermatogenesis* identified in our GO analysis. Red colour indicates upregulation in the active testis and blue colour upregulation in the regressed testis. The size of sepia circle is proportional to the number of deregulated genes it represents.

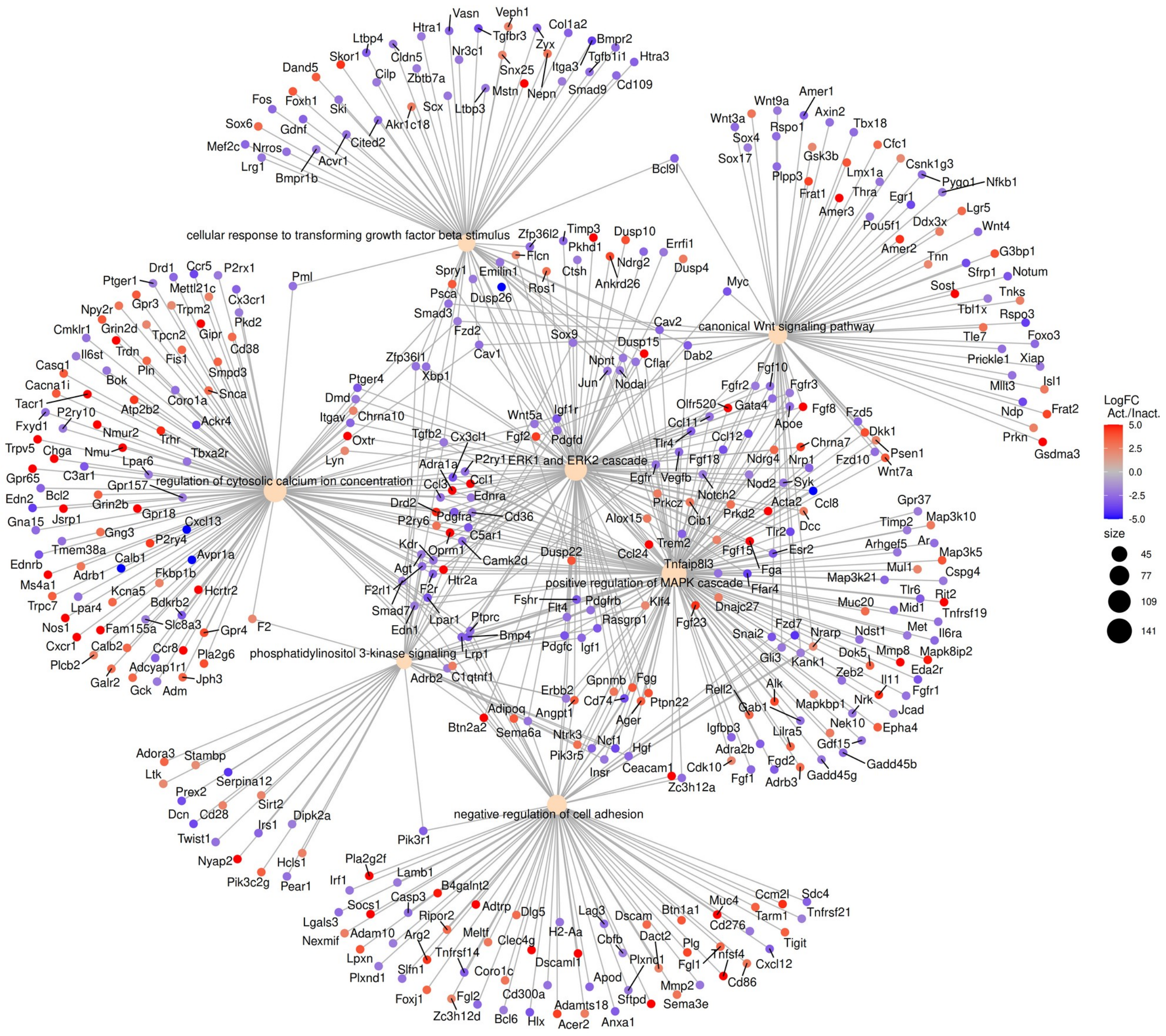

Supplementary Figure S2. Cnetplot of several significantly enriched GO terms of molecular pathways identified in our GO analysis. Red colour indicates upregulation in the active testis and blue colour upregulation in the regressed testis. The size of sepia circles is proportional to the number of deregulated genes they represent.

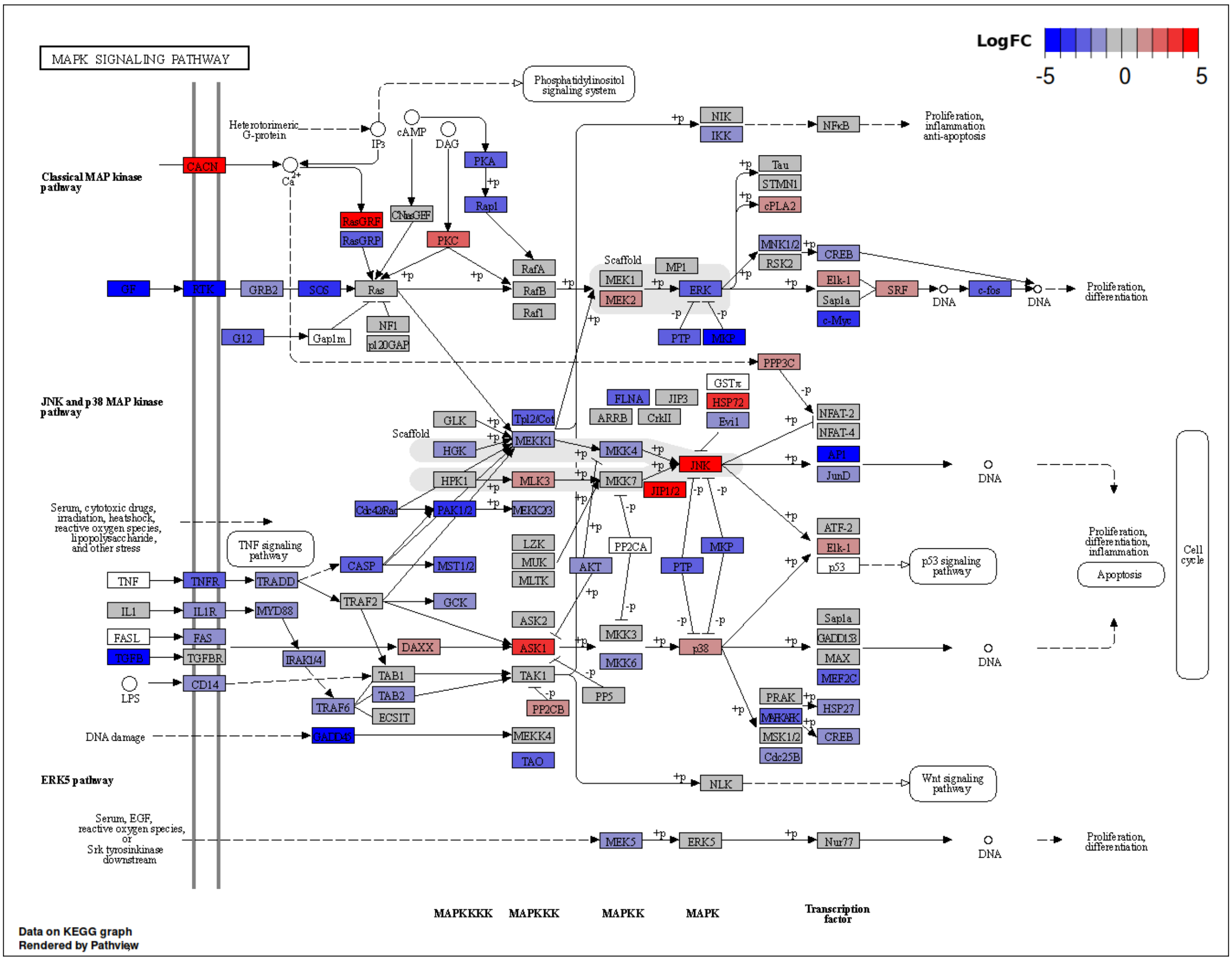

Supplementary Figure S3. Visualization of the differentially expressed genes of the MAPK signalling pathway between active and regressed testes of *M. duodecimcostatus.* Red colour indicates upregulation in the active testis and blue colour upregulation in the regressed testis.

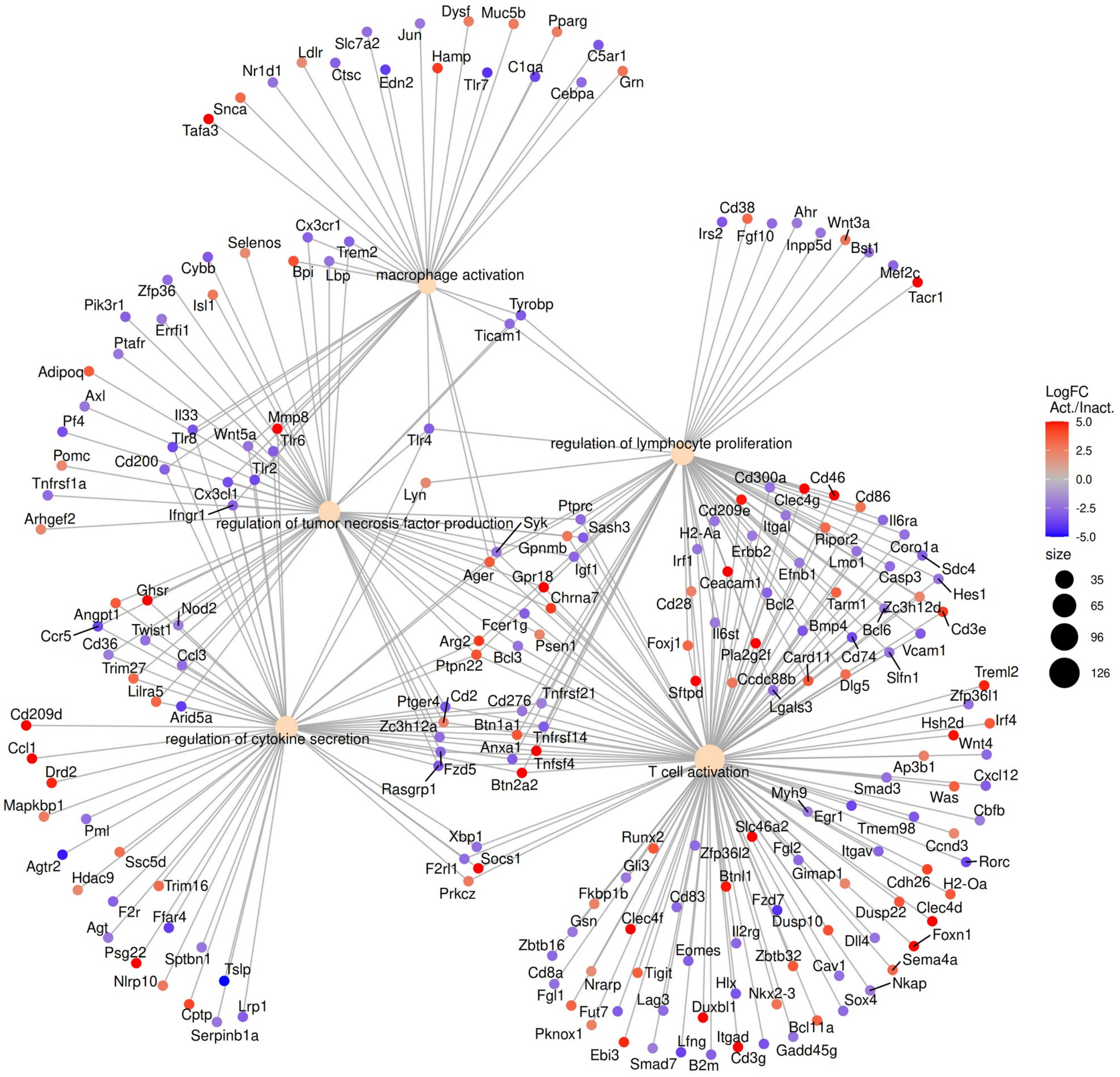

Supplementary Figure S4. Cnetplot of several significantly enriched GO terms associated with activation of the immune system identified in our GO analysis. Red colour indicates upregulation in the active testis and blue colour upregulation in the regressed testis. The size of sepia circles is proportional to the number of deregulated genes they represent.

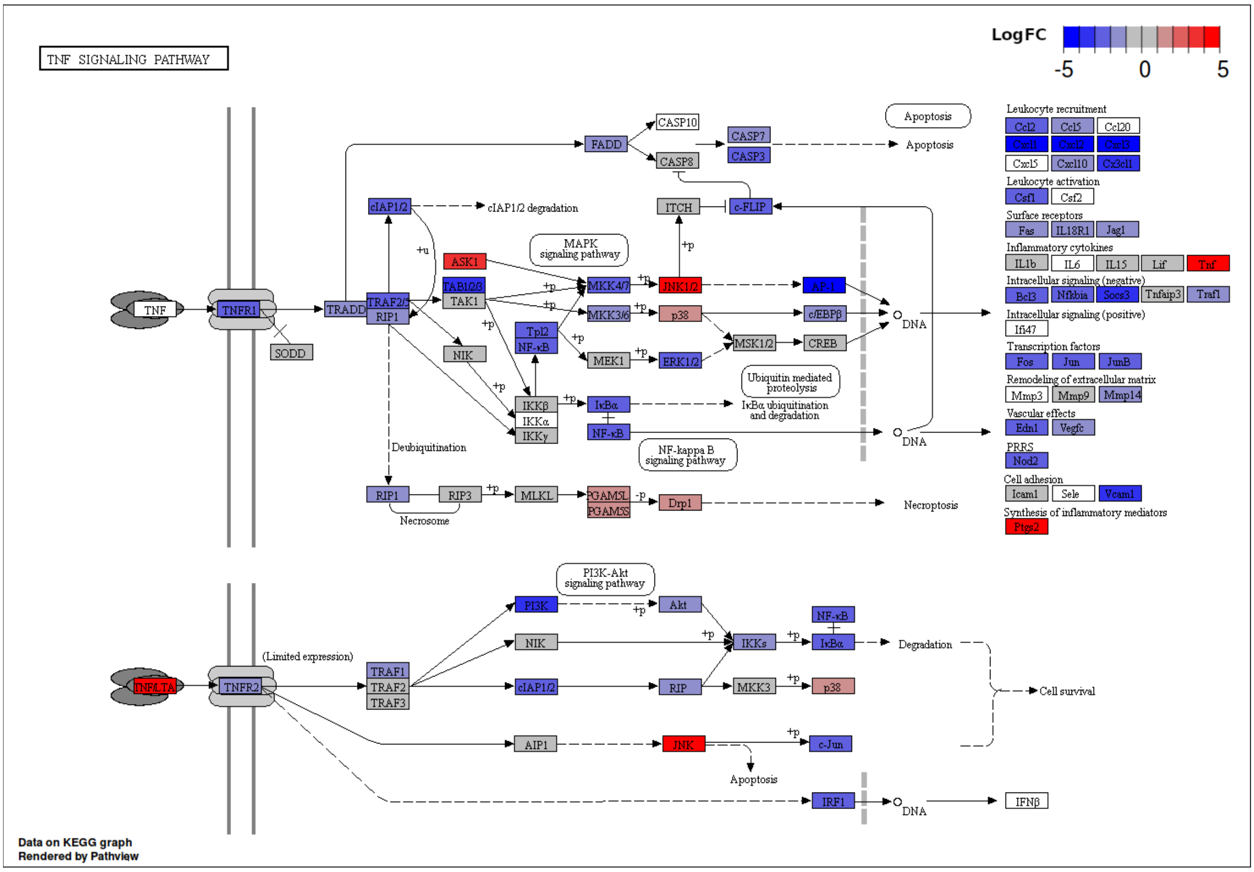

Supplementary Figure S5. Visualization of the differentially expressed genes of the TNF signalling pathway detected when comparing active and regressed testes of *M. duodecimcostatus*. Red colour indicates upregulation in the active testis and blue colour upregulation in the regressed testis.

Differential expression analysis

Transcriptome assembly

Transcriptome was assembled according to the Oyster River Protocol (ORP), a multi-assembler and kmer approach for de novo transcriptome assembly (MacManes 2018). To run the protocol we pulled the last docker container from macmaneslab/orp at DockerHub. In the ORP the quality of the assembled transcriptome was assessed by the Detonate and TransRate scores, as well as by BUSCO (Seppey, Manni, and Zdobnov 2019). As an indication of the high quality of the transcriptome, 87.09% of reads where mapped as proper pairs by BUSCO with a score of 95.7%.

Transcriptome annotation

The annotation was performed according to the trinotate pipeline (Bryant et al. 2017). Trinotate only works with Trinity assembled transcriptomes because the perl script **get_Trinity_gene_to_trans_map.pl** that makes the **get_Trinity_gene_to_trans_map** file uses regular expressions that match the Trinity style of naming transcripts in the **Trinity.fasta**. The usual recommended solution for using Trinotate with other assemblers is to rename the transcripts in the multifasta file to match the Trinity style or building a custom **gene_to_trans_map** file by other means. In order to simplify downstream analyses, our approach has been to use a custom nomenclature for the transcript names as actually they are a mixture of names from different aligners, so we modified the regular expressions in **get_Trinity_gene_to_trans_map.pl** to match our names.

Transcriptome Normalization

In this study we compare whole testis transcriptomes in two conditions, sexually active vs. inactive. The main cellular composition difference between both type of testes is the presence of the germ cells line only in the sexually active testis. This difference in cell composition will make that many genes expressed in germ cells will show as heavily overexpressed in sexually active testes and downstream analyses will be severely affected by these results, i.e. Gene Ontology terms

related with germ cells will be highly overrepresented in sexually active testes hiding the differential expression of genes in the somatic cell types that are shared by both tissues and are responsible for the regulation of spermatogenesis, specially concerning Sertoli cells. Thus, to normalize the data and make the transcriptomes comparable we decided to remove transcripts from the multiFASTA file that may belong from germ cells.

To select the transcripts that should be removed we used previously published cell signatures in single cell sequencing studies (Hermann et al. 2018; Green et al. 2018). These studies were performed in mouse, so all genes included in germ cells signatures belong from this species. The Trinotate report includes proteins from multiple species, as identified by BLASTX and BLASTP against protein databases. Thus, to identify the transcripts that should be removed we had to find the mouse orthologous proteins and genes corresponding to each of the multiFASTA entries.

We used a combined stategy to identify all possible mouse ortologous. We searched for ortologous mouse proteins and then for the genes that code for them or identified first the gene that code for a protein in a species and finding the ortologous mouse gene. We performed queries to the APIs of the Uniprot, ensembl and ensembl-compara databases starting from proteins IDs, gene symbols, entrez IDs or Ensembl IDs.

The Trinotate report, that includes the annotations of all the transcripts in the multiFASTA file, has 229172 entries corresponding to 154507 unique transcripts, and 114617 of them were mapped by Trinotate to proteins from multiple species. The combined strategy was able to identify the mouse ortologous genes of 113914 transcripts. These transcripts were included in a new processed FASTA file in which the mouse gene symbol and a transcript number were used to rename the transcripts. Transcripts with more than one possible mouse ortologous gene were named by concatenation of all the possible mouse gene symbols in order to preserve as much information as possible.

From this new FASTA files we removed all genes included in clusters 1-13 and 16 from (Hermann et al. 2018), belonging from different germ cell types, and those from spermatogonia, spermatocyte, round spermatid and elongating spermatid from (Green et al. 2018). 8282 transcripts from 7796 genes were discarded. The new purified FASTA file contained 105632 transcripts from 15807 genes.

To assess the effect of this normalization method we calculated the correlation coefficients between all possible sample pairs. The correlation coefficients between samples from the same category should be higher than those from different category. After normalization we expect a smaller difference between these coefficients as samples from different category must become more similar after removing the transcripts from the germ cell line. Accordingly, the mean correlation coefficient between samples from different category increased from ~0.5 to ~0.7 after normalization.

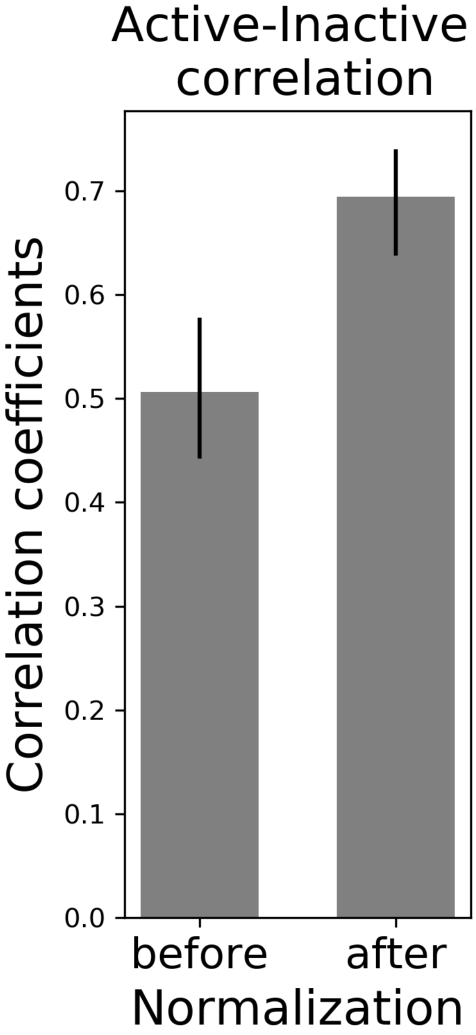

Figure 1: Correlations coefficients between sexually active and inactive testis samples at transcript level. The bars show the mean correlation coefficient. The vertical lines indicate the maximum and minimum correlation coefficients.

Transcript quantification

For transcript quantification we used Trinity with the ORP assembled transcriptome as reference (Grabherr et al. 2011; Haas et al. 2013). In order to run this pipeline with our transcript nomenclature we used our custom **get_Trinity_gene_to_trans_map.pl**, as previously described, and we also modified the regular expressions in **TrinityStats.pl** to accept non Trinity notations.

We checked the transcriptome quality as indicated in the software documentation. The statistics based on all transcript contig were:

```
Contig N10: 6366
Contig N20: 4737
Contig N30: 3736
Contig N40: 2953
Contig N50: 2322

Median contig length: 710
Average contig: 1306.27
Total assembled bases: 137983859
```

and based on only longest isoform per gene:

```
Contig N10: 8068
Contig N20: 6404
Contig N30: 5421
Contig N40: 4678
Contig N50: 4022

Median contig length: 2484
Average contig: 2950.40
Total assembled bases: 46636968
```

Both statistical results showed a high contig length.

As a more appropriated alternative, the N50 statistic may be limited to the top most expressed genes that represent a high percentage of the total normalized expression data. Plotting the Ex value against the ExN50 value showed a maximum near N85. The Ex85N50 statistic shows that 31028 genes have a transcript length of about 3.2kb, supporting the high quality of the transcriptome.

Each sample was aligned to this reference transcriptome with RSEM. (Li and Dewey 2011). We also performed a quality check of the samples according to the documentation of Trinity. Samples belonging from the same category (sexually active or inactive) showed a high correlation (~90%) at both the gene and transcript levels. Samples from different category showed a correlation of ~60% between samples at the gene level, and ~70% at the transcript level.

Principal component analyses showed that the main variable (active vs inactive)

explain 85.13% of the variability at the gene level, and 78.17% at the transcript level.

Differential expression analysis

DE analyses were performed with Trinity/RSEM according to the instructions in the Trinity documentation. Genes were considered as differentially expressed at $P < 0.001$ and $\log FC > 2$.

References

Bryant, Donald M., Kimberly Johnson, Tia DiTommaso, Timothy Tickle, Matthew Brian Couger, Duygu Payzin-Dogru, Tae J. Lee, et al. 2017. "A Tissue-Mapped Axolotl de Novo Transcriptome Enables Identification of Limb Regeneration Factors." *Cell Reports* 18 (January): 762–76. https://doi.org/10.1016/j.celrep.2016.12.063.

Grabherr, Manfred G., Brian J. Haas, Moran Yassour, Joshua Z. Levin, Dawn A. Thompson, Ido Amit, Xian Adiconis, et al. 2011. "Full-Length Transcriptome Assembly from RNA-Seq Data Without a Reference Genome." *Nature Biotechnology* 29 (May): 644–52. https://doi.org/10.1038/nbt.1883.

Green, Christopher Daniel, Qianyi Ma, Gabriel L. Manske, Adrienne Niederriter Shami, Xianing Zheng, Simone Marini, Lindsay Moritz, et al. 2018. "A Comprehensive Roadmap of Murine Spermatogenesis Defined by Single-Cell RNA-Seq." *Developmental Cell* 46 (September): 651–667.e10. https://doi.org/10.1016/j.devcel.2018.07.025.

Haas, Brian J., Alexie Papanicolaou, Moran Yassour, Manfred Grabherr, Philip D. Blood, Joshua Bowden, Matthew Brian Couger, et al. 2013. "De Novo Transcript Sequence Reconstruction from RNA-Seq Using the Trinity Platform for Reference Generation and Analysis." *Nature Protocols* 8 (August): 1494–1512. https://doi.org/10.1038/nprot.2013.084.

Hermann, Brian P., Keren Cheng, Anukriti Singh, Lorena Roa-De La Cruz, Kazadi N. Mutoji, I.-Chung Chen, Heidi Gildersleeve, et al. 2018. "The Mammalian Spermatogenesis Single-Cell Transcriptome, from Spermatogonial Stem Cells to Spermatids." *Cell Reports* 25 (November): 1650–1667.e8. https://doi.org/10.1016/j.celrep.2018.10.026.

Li, Bo, and Colin N. Dewey. 2011. "RSEM: Accurate Transcript Quantification from RNA-Seq Data with or Without a Reference Genome." *BMC Bioinformatics* 12 (August): 323. https://doi.org/10.1186/1471-2105-12-323.

MacManes, Matthew D. 2018. "The Oyster River Protocol: A Multi-Assembler and Kmer Approach for de Novo Transcriptome Assembly." *PeerJ* 6: e5428. https://doi.org/10.7717/peerj.5428.

Seppey, Mathieu, Mosè Manni, and Evgeny M. Zdobnov. 2019. "BUSCO: Assessing Genome Assembly and Annotation Completeness." *Methods in Molecular Biology (Clifton, N.J.)* 1962: 227–45. https://doi.org/10.1007/978-1-4939-9173-0_14.
